# Tumour cell density quantified by artificial intelligence is associated with differential benefit from irinotecan-based chemo-radiotherapy in locally advanced rectal cancer: a post-hoc study of the phase 3 ARISTOTLE trial

**DOI:** 10.1016/j.ebiom.2026.106397

**Published:** 2026-07-27

**Authors:** Zhuoyan Shen, Douglas Brand, Mikaël Simard, Nicholas P. West, Andre Lopes, Rubina Begum, Ying Zhang, Gary Royle, David Sebag-Montefiore, Charles-Antoine Collins Fekete, Maria A. Hawkins

**Affiliations:** a Department of Medical Physics and Biomedical Engineering, University College London, London, United Kingdom; b Department of Radiotherapy, University College London Hospitals NHS Foundation Trust, London, United Kingdom; c University of Leeds, Leeds, United Kingdom; d Cancer Research UK and UCL Cancer Trials Centre, London, United Kingdom; e Leeds Institute of Medical Research, University of Leeds, Leeds, United Kingdom

**Keywords:** Locally advanced rectal cancer, Tumour cell density, Chemoradiotherapy, Artificial intelligence, Digital pathology

## Abstract

**Background:**

Tumour cells and tumour-associated stroma are key components of the tumour microenvironment, and their interaction impacts disease progression and treatment resistance in rectal cancer. This study introduces a computational approach to quantify tumour cell density (TCD) within epithelial and stromal regions and assess whether treatment response differs according to TCD status in patients with locally advanced rectal cancer (LARC) undergoing neoadjuvant chemoradiotherapy (nCRT).

**Methods:**

The data of 414 ARISTOTLE trial (ISRCTN09351447) participants with available digitised pre-treatment biopsies were analysed in this study. We defined TCD as the proportion of tumour cells within the tumour and stroma tissues and quantified TCD using an AI framework applied to digitised haematoxylin and eosin-stained whole-slide images. The patients were stratified as TCD-high/TCD-low using a cut-off value of 0.5 (50% of tumour cells). The TCD status was combined with treatment arms [CRT (capecitabine + radiotherapy) and IrCRT (experimental capecitabine + irinotecan + radiotherapy)] to stratify the disease-free survival (DFS), overall survival (OS) and pathological complete response (pCR) rates.

**Findings:**

Among the patients analysed, 188 (45%) of 414 patients were classified as TCD-high and 226 (55%) as TCD-low. A significant treatment-TCD interaction was observed for both DFS (χ^2^ = 6.88, p = 0.009) and OS (χ^2^ = 10.61, p = 0.001). In the TCD-high subgroup, the use of IrCRT was associated with significantly longer DFS (HR = 0.57, 95% CI: 0.36–0.90, p = 0.014) and OS (HR = 0.50, 95% CI: 0.30–0.84, p = 0.008), along with a higher pCR rate (20 [23%] vs. 8 [11%], OR = 2.46, 95% CI: 1.01–5.98; p = 0.042 [p = 0.13 after adjustment for multiple testing]), compared to CRT. However, in the TCD-low subgroup, no significant difference in DFS was observed between treatment arms (HR = 1.29, 95% CI: 0.85–1.95, p = 0.22), while patients who received IrCRT exhibited a trend towards worse OS (HR = 1.55, 95% CI: 0.96–2.51, p = 0.07). No significant difference in pCR rates was observed (12 [14%] vs. 23 [22%], OR = 0.60, 95% CI: 0.28–1.29; p = 0.19).

**Interpretation:**

In this post-hoc, hypothesis-generating analysis of the ARISTOTLE trial, higher TCD was associated with differential outcomes after irinotecan-intensified neoadjuvant chemoradiotherapy compared with standard chemoradiotherapy. These findings support further evaluation of AI-derived TCD as a candidate predictive biomarker in independent retrospective and prospective cohorts.

**Funding:**

Cancer Research UK Radiation Research Network - Project Seed Funding (RRNPSF-Jan21/100001), Cancer Research UK ARISTOTLE sample collection grant (A18745), UK Research and Innovation Future Leadership Fellowship (No. MR/T040785/1) and the Radiation Research Unit at the Cancer Research UK City of London Centre Award (C7893/A2899).

**Research in context**

**Evidence before this study**

Prior to this study, a systematic review of PubMed and Cochrane databases was conducted to identify clinical trials on neoadjuvant chemoradiotherapy (nCRT) for locally advanced rectal cancer (LARC) and studies reporting the impact of tumour stroma ratio (TSR) and tumour cell density (TCD) between July, 2015, and July, 2025. 217 trials were found using the terms “locally advanced rectal cancer” AND “neoadjuvant chemoradiotherapy” AND “phase 3”. They reported that nCRT improves local control and increases the possibility of sphincter preservation in LARC, but survival benefits and the optimal criteria for patient selection remain areas of ongoing investigation. 23 articles were found using the terms “rectal cancer” AND “stroma” AND “chemoradiotherapy”. These studies highlighted that the stromal component was found to play a crucial role in tumour progression and resistance to therapy, and a greater proportion of stromal tissue is associated with poor prognosis in rectal cancer. However, inconsistency was observed in studies describing the impact on patients with LARC. We did not identify previous studies reporting the impact of TSR or TCD on patient stratification in cohorts treated by irinotecan nCRT.

**Added value of this study**

This study reports the impact of TCD in the context of irinotecan-based nCRT for patients with LARC. Although the addition of irinotecan did not yield a higher pathological complete response (pCR) rate, nor a survival advantage in the overall ARISTOTLE trial cohort, this study shows that patients with a high TCD phenotype derive significant benefit from irinotecan-based nCRT, including higher rates of pCR, longer DFS and OS. Meanwhile, this study applied artificial intelligence (AI) models to automatically identify TCD from digitised H&E-stained whole slide images, which is cost-efficient, scalable and is expected to be more robust compared to the manual estimation from selected regions.

**Implications of all the available evidence**

The results provide a potential stratification for future clinical trials to include TCD as a possible biomarker for selecting patients with LARC for irinotecan-intensified nCRT. This study also demonstrated the feasibility of deploying AI models on routine H&E-stained biopsies to quantitatively analyse the TCD, providing evidence and rationale for evaluating AI-derived TCD as a stratification factor in future studies. Future research should focus on validating the findings in larger, diverse retrospective and prospective cohorts and exploring the integration between TCD and other genomic and molecular markers to further refine patient selection and treatment planning in LARC.

## Introduction

Colorectal cancer (CRC) is the fourth most common and the second most fatal cancer in the UK.^1^ Rectal cancer diagnosed as clinical stage II or III is defined as locally advanced rectal cancer (LARC).^2^ Patients with LARC have a higher risk of recurrence, necessitating more intensive neoadjuvant treatment strategies than for patients with early-stage rectal cancer.^3^ A recent shift in LARC management involves the adoption of total neoadjuvant therapy (TNT), where chemotherapy and radiotherapy are administered before total mesorectal excision (TME).^4^ Trials investigating the changes in the TNT regimes have been published with evidence that might change clinical practice.^5^^,^^6^ Despite these advances, current approaches have largely focused on altering treatment sequencing rather than tailoring treatment selection based on tumour biology. The emerging techniques, including artificial intelligence (AI), digital pathology and whole genome sequencing (WGS), offer the opportunity to develop molecular signatures for precise treatment selection.

The tumour microenvironment (TuME) comprises innate and adaptive immune cells, tumour vasculature, fibroblasts, and tumour cells embedded within the extracellular matrix (ECM). Together, these components play a pivotal role in shaping tumour phenotype, driving evolutionary dynamics, and influencing response to therapy. The prognostic significance of the TuME is well-established across multiple cancer types,^7^ with increasing evidence highlighting the importance of interactions between tumour and stromal cells.^8^ The deposition of stromal cells can create a protective environment for the tumour: higher stromal density is linked to faster tumour progression, metastatic spread, and treatment resistance.^9^

One widely studied histopathological summary of tumour–stroma composition is the tumour-to-stroma ratio (TSR), typically defined as the proportion of tumour area relative to stromal area within a representative tumour region. A lower tumour-to-stroma ratio (TSR) in pre-treatment biopsies is associated with worse OS and DFS in various solid epithelial tumours, including CRC.^10^ However, TSR assessment is often performed by visual estimation on H&E-stained sections, and studies have reported interobserver variability among pathologists in different career stages.^11^^,^^12^ Tumour cell density (TCD) provides a cell-level measure of tumour content by quantifying the relative abundance of tumour cells within defined regions. Previous studies calculated TCD using manually classified tumorous or stromal points from the specimens to derive more precise TSR-like measures.^13^^,^^14^ However, manual TCD assessment is time-consuming and impractical for large-scale studies or routine clinical deployment. Recent studies have demonstrated that analysing digitised pathology slides using AI-based approaches can effectively automate this measurement.^15^

In this study, we deployed an AI framework to automatically quantify TCD from pre-treatment biopsy whole slide images of participants enrolled in the ARISTOTLE trial (ISRCTN09351447).^16^ The ARISTOTLE trial is a phase III clinical trial investigating whether the addition of a second drug (irinotecan) to the standard neoadjuvant chemoradiotherapy (nCRT) of long-course capecitabine and radiotherapy improves outcomes for patients with MRI-defined LARC. While the trial demonstrated no significant survival benefit for the irinotecan arm, increased treatment-related toxicity was observed, highlighting the need for better patient stratification. We considered that more aggressive and chemo-resistant TCD-low (high proportion of stroma) tumours would respond less well to treatment intensification. Specifically, we hypothesised that patients with TCD-high tumours would derive greater benefit from irinotecan-based chemoradiation, with improved disease-free survival (DFS) and overall survival (OS). If validated, TCD could be further evaluated as a candidate biomarker to identify patients most likely to benefit from irinotecan intensification in future nCRT strategies.

## Methods

### Data source

This study is a hypothesis-generating post-hoc analysis of the ARISTOTLE trial and was not conducted according to a pre-specified protocol. Participants were recruited between October 2011 and July 2018, and the present study was conducted from January 2023 to September 2024. Two treatment arms were presented in the trial: A) Standard arm (CRT) - capecitabine 900 mg/m^2^ orally twice daily Mon-Fri for five weeks with radiotherapy 45Gy in 25 fractions; B) Experimental arm (IrCRT) - irinotecan 60 mg/m^2^ intravenous once weekly weeks 1–4 and capecitabine 650 mg/m^2^ orally twice daily Mon-Fri for five weeks with radiotherapy 45Gy in 25 fractions. The data used for this study comprised participant clinical data, follow-up reports, and digitised WSIs of haematoxylin and eosin (H&E)-stained pre-treatment biopsies (Table S1). Diagnostic years range from 2011 to 2018. The follow-up time is up to five years. Sex data were collected from clinical records as documented by the treating clinical teams. Targeted next-generation sequencing (NGS) data from an 80-gene colorectal cancer driver panel were available for a sub-cohort of 242 patients. For downstream analyses, KRAS and TP53 were prioritised because they were the most frequently altered genes in this dataset, with mutation prevalence of 55.96% and 70.25%, respectively.

### Study design

**Fig. 1:**
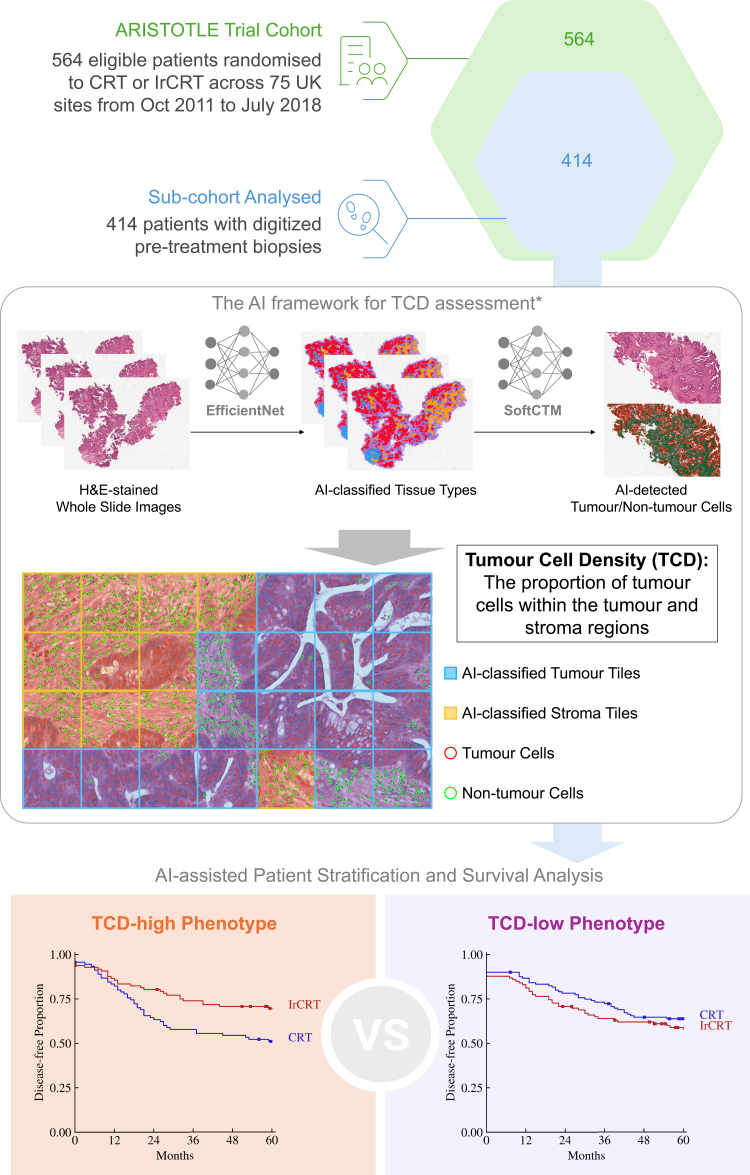
**Workflow of the study.** The AI framework consists of a tissue classifier, an EfficientNet trained on the NCT-CRC-HE-100K dataset, and a cell detector, the SoftCTM. The tissue classifier segments whole-slide images into nine types of colorectal tissues, including tumour and stroma, while SoftCTM detects and classifies individual cells as tumour or non-tumour. The tumour cell density in this study is defined as the proportion of detected tumour cells within the AI-classified tumour and stroma regions. Patients were stratified into TCD-high and TCD-low groups using a cutoff of 0.5.

Fig. 1 illustrates the workflow of this study. A subset of 414 patients with available pre-treatment biopsy samples was selected for analysis. We evaluated both the prognostic and predictive relevance of the AI-derived TCD. The prognostic effect was defined as whether TCD was associated with clinical outcomes irrespective of treatment assignment, whereas the predictive effect was defined as whether TCD modified treatment benefit. DFS and OS are used as the primary outcome measures in this study. Pathological complete response (pCR) was analysed as the secondary outcome measure. pCR data was assessed from the resection specimen and was therefore unavailable in patients who did not undergo surgery or had no evaluable surgical pathology (N = 59). Accordingly, analyses of pCR were restricted to the remaining 355 of 414 patients with available resection pathology.

### AI framework

An AI framework consisting of a tissue classifier and a cell detector was developed and deployed to automatically identify the TCD of 414 biopsies. The AI framework consists of a tissue classifier, an EfficientNet^17^ trained on the NCT-CRC-HE-100K dataset,^18^ and a pre-trained cell detector, SoftCTM.^19^ AI model development and digital pathology data analyses were conducted blinded to clinical outcomes.

The EfficientNet model was trained on a single NVIDIA GeForce RTX 3090 GPU over 100 epochs, with a batch size of 256. The AdamW optimiser with an initial learning rate of 0.0001 was applied. Spatial data augmentation included random horizontal flipping (p = 0.5), and colour augmentation was used during training. Specifically, RGB histology images were first separated into haematoxylin and eosin (H&E) channels using the stain deconvolution.^20^ Controlled perturbations^21^ were then introduced to the stain concentrations, following a uniform sampling (σ = 0.14) applied to the H&E channels prior to RGB reconstruction. Both the NCT-CRC-HE-100k dataset and the ARISTOTLE cohort consisted of colorectal cancer H&E-stained histopathology slides scanned at comparable magnifications (20×). To further harmonise the datasets, image resizing based on pixel spacing was applied to ensure consistent spatial resolution between training and inference datasets. Detailed dataset splits (training, validation, testing) and the corresponding performance metrics for tumour and stroma are provided in Table S3.

The tissue classifier segments whole-slide images into nine types of colorectal tissues, including tumour and stroma, while SoftCTM detects and classifies individual cells as tumour or non-tumour. The applicability of SoftCTM to colorectal cancer tissue has previously been independently validated in a large-scale study including 1097 colorectal cancer cases across different institutions and scanning pipelines.^22^ The study demonstrated that SoftCTM achieved robust and reproducible single-cell level quantification across these heterogeneous datasets.

For evaluating the quality of the proposed AI-based TCD identification method, we applied the framework to two datasets with TCD manually annotated by pathologists.

1. 116 WSIs, including both biopsy and resection samples, collected from a published study^23^;
2. 100 resection samples from the ARISTOTLE cohort that had been annotated for an independent ongoing study.

AI-based TCD quantification was performed retrospectively on these slides, and the completed annotations were reused as a reference standard for concordance assessment. The agreement between AI-detected tumour regions and pathologist-annotated tumour-associated regions was also evaluated in the 100 ARISTOTLE samples. Because the pathologist's annotations were intentionally broad and included surrounding tumour-associated tissues, precision was used for assessing the specificity of AI-predicted tumour regions. In addition, recall was evaluated for AI-detected tumour-associated tissues after excluding background and normal epithelial regions.

### Definition of the TCD and stratified groups

In this study, we define the TCD as the proportion of tumour cells relative to all classifiable cells (tumour + non-tumour) in the neoplastic epithelial and tumour-associated stromal region detected by the AI model. Morphologically normal tissue was excluded.

We stratify the patients into two groups based on the TCD in their pre-treatment biopsies.

• TCD-high: Patients with TCD higher than or equal to 0.5
• TCD-low: Patients with TCD lower than 0.5

The cut-off value of 0.5 was selected as the median AI-derived TCD in the overall cohort and was not optimised on outcome. This threshold also aligns closely with thresholds used in previous studies evaluating the prognostic and predictive value of TCD or TSR in colorectal cancer.^14^^,^^15^^,^^24^

### Ethics

Ethical approval for the ARISTOTLE trial was granted by the NHS Health Research Authority (HRA) (REC reference: 10/H0706/65, IRAS ID: 5406). Written informed consent was obtained from all participants. The study adheres to the 2024 World Medical Association Declaration of Helsinki.

### Statistics

As this study represents a post-hoc analysis of the ARISTOTLE trial, all cases with available pre-treatment biopsy specimens were included (N = 414). Concordance between AI-derived and manually assessed TCD was evaluated using Lin's concordance correlation coefficient (CCC) with 95% CI. Agreement was further examined using Bland–Altman plots to visualise bias and limits of agreement. Scatter plots were provided for descriptive purposes only and not used to formally assess concordance. Chi-squared tests were used for the differentiation analysis of categorical baseline characteristics. Kaplan–Meier estimation was used for survival analysis, and log-rank testing was performed to discriminate the survival curves of different subgroups. Hazard Ratios (HR) with a 95% confidence interval (CI) were given by the Cox proportional hazard model without additional adjustment for baseline clinicopathological covariates.

To assess the interaction between TCD status and treatment arm on DFS and OS, we compared nested Cox proportional hazards models with and without an interaction term (TCD × treatment arm). Model performance was assessed using Akaike Information Criterion (AIC) and log-likelihood values. A likelihood ratio test was used to determine the statistical significance of the interaction term. For the analysis of pCR, odds ratios (OR) with 95% CI were calculated to compare the probability of response between treatment arms. A two-sample Z-test for proportions was used to evaluate the statistical significance of differences in pCR rates in the overall cohort and within TCD-defined subgroups.

### Role of funders

The ARISTOTLE trial sample collection and slide scanning were funded by Cancer Research UK (A18745). The other funders played no direct roles in study design, data collection, data analysis, interpretation, or writing of the report. None of the authors received compensation from the funder or any other entity for writing this article.

## Results

### Tumour cell density identified by AI

Figure S1 shows representative examples comparing AI-classified tissue types with corresponding manual annotations. The AI tissue classifier achieved a precision of 0.873 for tumour region identification and a recall of 0.984 for tumour-associated tissues.

**Fig. 2:**
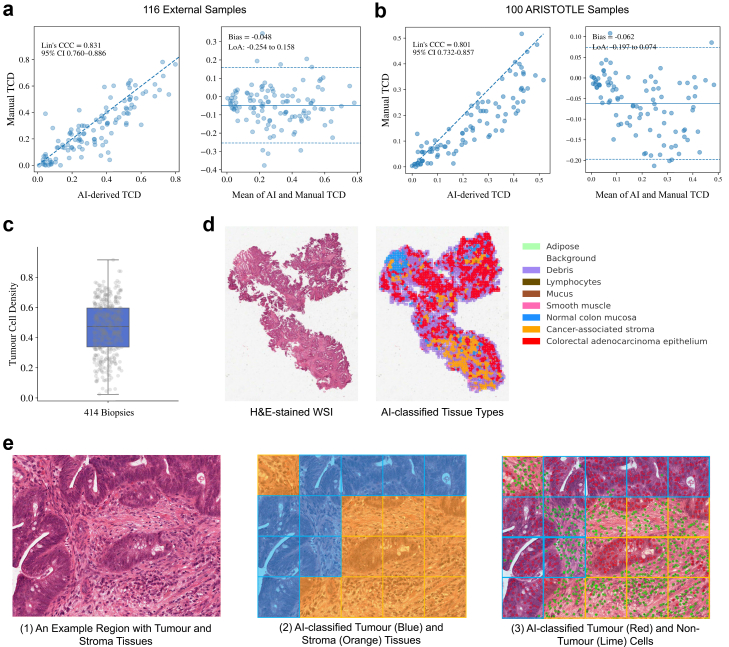
**Illustration of the quality of the AI-identified TCD.** a. Scatter plot (left) demonstrating concordance between AI-derived and manually assessed TCD in 116 externally collected WSIs, with a Lin's concordance correlation coefficient (CCC) of 0.831 (95% CI: 0.760–0.886). Bland–Altman plot (right) showing the difference between manual and AI-derived TCD measurements against their mean values. The solid line represents the mean bias (−0.048), and the dashed lines indicate the 95% limits of agreement (−0.254 to 0.158). b. Scatter plot (left) demonstrating concordance between AI-derived and manually assessed TCD in 100 ARISTOTLE resection WSIs, with a CCC of 0.801 (95% CI: 0.732–0.857). Bland–Altman plot (right) showing the difference between manual and AI-derived TCD measurements against their mean values. The solid line represents the mean bias (−0.061), and the dashed lines indicate the 95% limits of agreement (−0.197 to 0.074). c. Distribution of AI-identified TCD across 414 biopsy samples. d. An example H&E-stained WSI and its corresponding AI-based tissue-type classification. e. AI detected tumour (red)/non-tumour (green) cells in a tumour-stroma interaction region at 20 × magnification.

As illustrated in Fig. 2a, in the 116 externally collected WSIs, AI-derived TCD demonstrated strong concordance with manually assessed TCD using Lin's concordance correlation analysis (CCC = 0.831, 95% CI: 0.760–0.886). Bland–Altman analysis showed a mean bias of −0.048 (manual lower than AI), with 95% limits of agreement ranging from −0.254 to 0.158. Similarly, in the 100 ARISTOTLE resection WSIs (Fig. 2b), Lin's concordance correlation analysis demonstrated strong agreement between AI-derived and manually assessed TCD (CCC = 0.801, 95% CI: 0.732–0.857). Bland–Altman analysis demonstrated a mean bias of −0.061 (manual lower than AI), with 95% limits of agreement from −0.197 to 0.074. Using median cut-offs to stratify cases into TCD-high and TCD-low groups, AI-derived and manual TCD classifications showed strong categorical agreement (93%), with a Cohen's kappa coefficient of 0.854 and Fisher's exact test p < 0.0001.

Comparative assessment showed no significant differences in baseline clinical characteristics or survival outcomes between the sub-cohort analysed in this study and the full ARISTOTLE trial population (Tables S2). Across the entire cohort, TCD values ranged from 0.02 to 0.91, with a median of 0.472 and a standard deviation of 0.174 (Fig. 2c). Based on TCD stratification, 188/414 (45%) patients were stratified as TCD-high, and 226/414 (55%) patients were stratified as TCD-low. Examples of tissue classification results and tumour cell detection results are illustrated in Fig. 2d and e, respectively.

### Patient characteristics and clinical outcomes

Table 1 summarises the distribution of patients grouped by baseline characteristics and TCD. Table 2 shows the clinical outcomes of patients stratified by TCD status. The three-year disease-free rates and survival rates are 68% and 87%, respectively. The five-year disease-free rates and survival rates are 61% and 76%, respectively.

**Table 1:** Baseline characteristics of patients stratified by TCD status.

Characteristic	Overall (%) N = 414	TCD-high (%) N = 188	TCD-low (%) N = 226	*p*^a^
Age (years)				
≤60	192 (46)	87 (46)	105 (46)	>0.999
>60	222 (54)	101 (54)	121 (54)	
Sex				
Female	143 (34)	68 (36)	75 (33)	0.60
Male	271 (66)	120 (64)	151 (67)	
Trial arm				
CRT	211 (51)	91 (48)	120 (53)	0.39
ICRT	203 (49)	97 (52)	106 (47)	
MRI T stage				
T2	23 (6)	11 (6)	12 (5)	0.18
T3	318 (78)	151 (81)	167 (75)	
T4	68 (17)	24 (13)	44 (20)	
Missing	5			
MRI N stage				
N0	100 (24)	50 (27)	50 (22)	0.39
N1	186 (46)	78 (42)	108 (49)	
N2	123 (30)	58 (31)	65 (29)	
Missing	5			

a p—values from the Chi-squared test.

**Table 2:** Clinical outcomes of patients stratified by TCD status.

Outcome	Overall (%) N = 414	TCD-high (%) N = 188	TCD-low (%) N = 226	*p*^a^
pCR				
Non-Responder	292 (82)	135 (83)	157 (82)	0.80
Complete response	63 (18)	28 (17)	35 (18)	
Missing	59			
DFS in month (median [Q1, Q3])	61.5 [20.4, 84.4]	61.5 [19.4, 84.0]	61.5 [21.8, 85.5]	0.78
OS in month (median [Q1, Q3])	66.0 [54.4, 96.1]	65.1 [55.2, 90.3]	67.3 [54.4, 99.0]	0.44

a P-values for pCR were derived from two-sample tests for equality of proportions (z-test). P-values for DFS and OS were derived from log-rank tests.

### Interaction between TCD and nCRT regimens

The treatment-TCD interaction was significant for both DFS (HR: 0.44 [0.24–0.82], p = 0.009) and OS (HR: 0.31 [0.16–0.64], p = 0.001) in Cox proportional hazards interaction models. For DFS, inclusion of the interaction term improved model fit, reducing the AIC from 1936.62 to 1931.73 and increasing the log-likelihood from −966.31 to −962.87. A likelihood ratio test confirmed that the interaction was statistically significant (χ^2^ = 6.88, p = 0.009). Similarly, for OS, the interaction model showed improved fit (AIC: 1457.47 vs 1448.86; log-likelihood: −726.73 vs −721.43), with the likelihood ratio test again indicating a significant interaction (χ^2^ = 10.61, p = 0.001).

In sensitivity analyses restricted to the sub-cohort with available DNA mutation data, the treatment-TCD interaction remained significant for both DFS (HR: 0.39 [0.17–0.91], p = 0.029) and OS (HR: 0.30 [0.11–0.81], p = 0.017) after adjustment for KRAS and TP53. The interaction model also showed improved fit for DFS (AIC: 951.81 vs 949.04; log-likelihood: −471.91 vs −469.52) and OS (AIC: 711.24 vs 707.42; log-likelihood: −351.62 vs −348.71), with the likelihood ratio test again indicating a significant interaction for DFS (χ^2^ = 4.77, p = 0.029) and OS (χ^2^ = 5.82, p = 0.016).

### The impact of TCD and nCRT regimens on DFS and OS

**Fig. 3:**
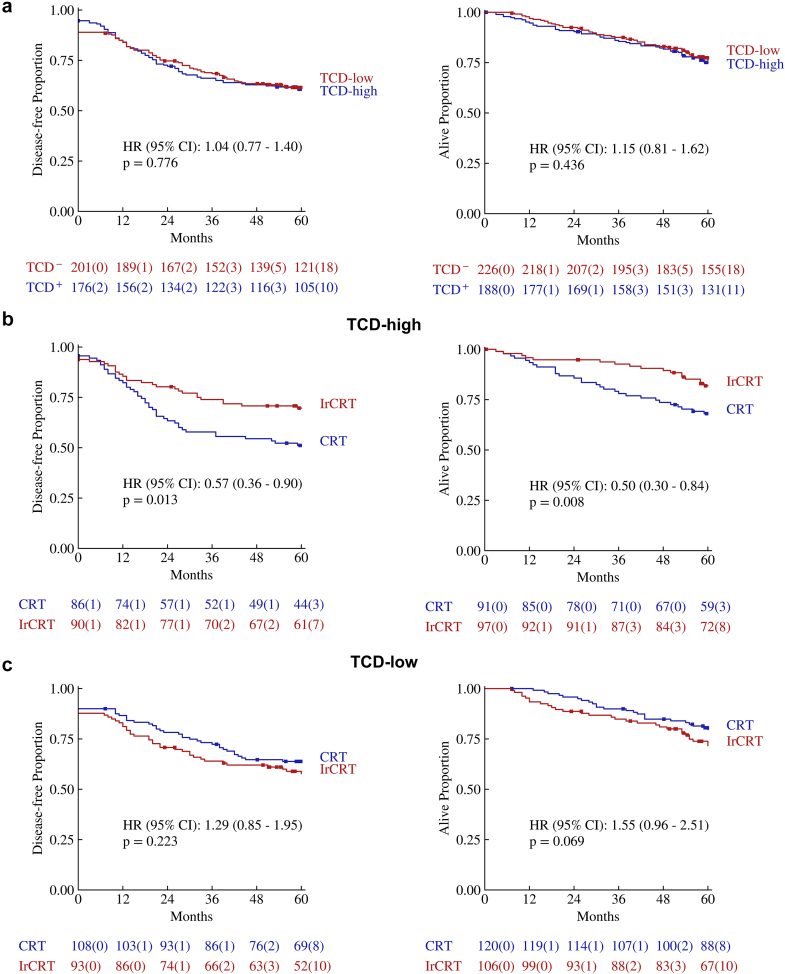
**Survival difference between TCD-high and TCD-low patients, and between IrCRT and CRT in the subgroups stratified by TCD.** Kaplan–Meier curves are shown for disease-free survival (DFS, left panels) and overall survival (OS, right panels). Hazard ratios (HRs) with 95% confidence intervals (CIs) and two-sided log-rank p-values are displayed within each panel. Numbers below the x-axis indicate the number of patients at risk at each time point; values in brackets represent the number of patients censored. a. The Kaplan–Meier curves of DFS (left) and OS (right) between TCD-high and TCD-low patients. b. The Kaplan–Meier curves of DFS (left) and OS (right) between IrCRT and CRT arms in the TCD-high group. c. The Kaplan–Meier curves of DFS (left) and OS (right) between IrCRT and CRT arms in the TCD-low group.

Between the patients in the TCD-low and TCD-high subgroups, Cox proportional hazards regression demonstrated no significant difference was observed in DFS (HR = 1.04, 95% CI: 0.77–1.40, p = 0.78) or OS (HR = 1.15, 95% CI: 0.81–1.62, p = 0.44) (Fig. 3a). Within the IrCRT group, patients in the TCD-high subgroup showed a non-significant trend toward longer DFS (HR = 0.68, 95% CI: 0.44–1.07, p = 0.10) and OS (HR = 0.63, 95% CI: 0.37–1.06, p = 0.08) compared to patients in the TCD-low subgroup. In contrast, within the CRT group, patients in the TCD-high subgroup had significantly shorter DFS (HR = 1.54, 95% CI: 1.02–2.33, p = 0.04) and OS (HR = 2.04, 95% CI: 1.26–3.29, p = 0.003) compared to patients in the TCD-low subgroup (Figure S2).

In the whole cohort, IrCRT did not significantly alter DFS (HR = 0.89, 95% CI: 0.66–1.21, p = 0.46) and OS (HR = 0.92, 95% CI: 0.65–1.29, p = 0.62) (Figure S3). Notably, within the TCD-high group, IrCRT conferred significantly improved DFS (HR = 0.57, 95% CI: 0.36–0.90, p = 0.014) and OS (HR = 0.50, 95% CI: 0.30–0.84, p = 0.008) compared to CRT (Fig. 3b). However, within the TCD-low group, no evidence of differential treatment effect in DFS was observed (HR = 1.29, 95% CI: 0.85–1.95, p = 0.22), while patients who received IrCRT showed a non-significant trend toward shorter OS (HR = 1.55, 95% CI: 0.96–2.51, p = 0.07), although the trend did not reach statistical significance (Fig. 3c).

### The impact of TCD and nCRT regimens on pCR

Table 3 presents the distribution of pCR across treatment arms, stratified by TCD group. Data on pCR were missing for 59 patients (14%), and analyses were based on the remaining 355/414 patients with complete outcome data.

**Table 3:** Distribution of pCR by Treatment Arm and TCD Group.

Subgroup	pCR	Overall (%) N = 414	CRT (%) N = 211	IrCRT (%) N = 203	OR (95% CI)	p-value (unadj/adj)
TCD-high (N = 188)	Non-responder	135 (83)	67 (89)	68 (77)	2.46 (1.01–5.98)	0.042/0.13
	Complete response	28 (17)	8 (11)	20 (23)		
	Missing	25				
TCD-low (N = 226)	Non-responder	157 (82)	84 (78)	73 (86)	0.60 (0.28–1.29)	0.19/0.56
	Complete response	35 (18)	23 (22)	12 (14)		
	Missing	34				
Whole cohort (N = 414)	Non-responder	292 (82)	151 (83)	141 (82)	1.11 (0.64–1.91)	0.72/>0.999
	Complete response	63 (18)	31 (17)	32 (18)		
	Missing	59				

Odds ratios (OR) with 95% confidence intervals (CI) were estimated using logistic regression (unadjusted). Unadjusted and Bonferroni-adjusted p-values are shown.

Overall, logistic regression analysis demonstrated no significant difference in pCR rate was observed between the CRT and IrCRT groups (31 [17%] vs. 32 [18%]; OR = 1.11, 95% CI: 0.64–1.91; two-sample Z-test p = 0.72). Similarly, in the TCD-low group, there were no significant differences between CRT and IrCRT in pCR rates (OR = 0.60, 95% CI: 0.28–1.29; two-sample Z-test p = 0.19), although a slightly lower pCR rate was noted in patients receiving IrCRT (12 [14%] vs. 23 [21%]). In contrast, the TCD-high subgroup demonstrated a more favourable response trend with IrCRT, where a significantly greater proportion of patients achieved pCR compared to those receiving CRT (20 [23%] vs. 8 [11%]; OR = 2.46, 95% CI: 1.01–5.98; two-sample Z-test p = 0.042) compared to CRT. However, after adjustment for multiple testing, this association was no longer statistically significant.

## Discussion

In this study, an AI-based digital pathology framework to quantify TCD from H&E-stained WSIs was developed and applied to the pre-treatment biopsies from a sub-cohort of 414 patients from the randomised phase III ARISTOTLE trial (total n = 564) to evaluate both the prognostic association of baseline TCD with outcomes irrespective of treatment, and the predictive value of TCD as a modifier of benefit from irinotecan-intensified nCRT. The framework applies deep-learning models for tissue compartment identification (tumour vs stroma) and tumour cell detection, enabling whole-slide estimation of TCD at scale. In the ARISTOTLE trial cohort, we observed heterogeneity of treatment effect across TCD strata for DFS, OS and pCR, supporting TCD as a candidate biomarker for treatment selection, subject to external clinical validation.

Consistent with the primary trial report, the addition of irinotecan to standard neoadjuvant chemoradiotherapy (IrCRT) did not significantly improve DFS or OS in the sub-cohort analysed in this study. This highlights the need for predictive biomarkers to identify patients who may benefit from treatment intensification. The principal finding of this study is a predictive signal: stratification by baseline TCD indicated heterogeneity of treatment effect, supported by a significant treatment-TCD interaction for both DFS and OS. This interaction remained significant after adjustment for KRAS and TP53 in the mutation-available subset. Patients with high TCD who received IrCRT experienced clinically meaningful improvements in DFS and OS and were more likely to achieve pCR, suggesting that tumours with a lesser stromal component may respond more favourably to irinotecan. Previous studies have demonstrated a relationship between the TCD or TSR and response to nCRT in LARC. Strous et al.^25^ reported that a high stroma ratio in biopsy specimens was associated with a lower likelihood of pCR, while Liang et al.^26^ found that TSR could effectively distinguish non-responders to nCRT. The finding in our study aligns with the hypothesis that abundant tumour-associated stroma may contribute to chemoresistance, and importantly, extends previous research by demonstrating this effect within the context of an experimental treatment arm in a randomised clinical trial.

Notably, in the TCD-low group, a trend toward worse OS with IrCRT was observed. This may be attributable to irinotecan-associated toxicity outweighing therapeutic benefit in patients with limited tumour cellularity. Indeed, the ARISTOTLE trial reports a higher rate of Grade ≥3 adverse events and worse radiotherapy and capecitabine compliance in the IrCRT group. However, although IrCRT did not improve outcomes across the overall trial cohort, our results suggest TCD quantified from pre-treatment biopsies may serve as a useful biomarker for identifying patients with LARC who could benefit from the addition of irinotecan to standard nCRT.

In contrast, the prognostic effect of TCD was limited in our study. No association between the TCD and DFS or OS was found in the cohort analysed. The ratio between tumour and stroma has been reported as a prognostic marker in some diseases, although the impact was less clear in CRC. Sullivan et al.^27^ reviewed the published studies discussing the prognostic value of TSR in CRC and found that the association was not observed in studies with shorter follow-up periods but became more significant in long-term outcome prediction. A recent meta-analysis reported that the association between the TSR and outcomes was only significant in the stage II and mixed stages of patients with CRC^28^ Additionally, van Wyk et al. found that the relationship between the TSR and cancer-specific survival was only found in T3 primary operable CRC.^29^ Less evidence has been reported for patients with LARC receiving nCRT. In our study, the impact of the two regimes of nCRT may lead to an unclear pattern of association between the TCD and outcomes.

As the selection of patients with LARC for personalised therapeutic strategies gains importance—driven by the shift towards total neoadjuvant approaches and watch-and-wait strategies—the implementation of rapid, biopsy-based biomarkers to personalise treatment is of paramount importance. The pressing need for reliable biomarkers to guide personalised treatment planning in LARC is highlighted by the ARISTOTLE trial, which shows no evidence of a benefit from the addition of irinotecan to standard CRT. Several post-hoc studies^30^, ^31^, ^32^ involving the ARISTOTLE cohort have already identified candidate biomarkers, such as gene expression signatures and consensus molecular subtypes, that may help predict which patients are more likely to achieve a pCR under standard CRT. However, the identification of specific subgroups that derive survival benefit from the addition of irinotecan remains unresolved.

The findings from this study provide the rationale to evaluate TCD as a biomarker to guide personalised treatment strategies in LARC. Given that irinotecan is associated with increased toxicity, including diarrhoea, lymphopenia, and neutropenia, identifying patients unlikely to respond based on low TCD could help avoid unnecessary treatment-related burden.

A key strength of this work is the deployment of an AI framework to characterise TCD, achieving near real-time performance (approximately 2 min per slide). The advantages of our approach include high efficiency, robustness, and reproducibility, addressing known issues with inter-observer variability among pathologists in distinguishing tumour from stromal cells. Traditional TCD assessment relies on visual inspection of selected regions under a microscope, often requiring manual counting of hundreds of cells, a time-consuming process that is not scalable to large datasets. For instance, the TCD method defined by West et al. involved manual estimation from 9 mm^2^ tissue regions, with an average processing time of 20 min per slide.^14^

Another strength is the cell-level nature of the proposed metric. Although previous studies have reported using AI and digital pathology in quantifying the tumour and stroma proportions,^33^ tissue tile-based classification may lack precision, as tumour tiles can include stromal cells and vice versa, limiting the granularity of TSR estimates. We also observed that tumour and stroma tiles classified by AI may contain a mixture of the two components (Fig. 2d), thereby limiting the accuracy of TSR estimation. To overcome this limitation, our proposed framework integrates both tissue classification and cell detection, allowing TCD to be quantified at the cell level from the WSIs.

Several limitations should be acknowledged. First, this is a post-hoc translational analysis of a trial sub-cohort, and the predictive signal observed for irinotecan intensification requires independent confirmation in external cohorts. Validation on independent LARC trial cohorts, such as CinClare,^34^^,^^35^ which investigated the benefit of irinotecan-based nCRT, would strengthen our findings. Second, prospective clinical validation is needed. A stratified clinical trial design, where patients are assigned to treatment arms based on biopsy-derived TCD levels, would be a critical next step to confirm the predictive utility of this biomarker and evaluate its impact on long-term outcomes and treatment-related toxicity. Third, although the proposed AI pipeline enabled scalable whole-slide quantification and showed strong agreement with manually annotated TCD in an independent external cohort, further methodological refinement may improve measurement precision. Future work will explore foundation-model-based feature extraction to enhance tissue compartment classification. Finally, molecular and genomic correlates were not comprehensively evaluated. Microsatellite instability (MSI) status was available for 242 of 414 patients; however, all cases with available data were microsatellite stable. Future studies should incorporate more detailed molecular profiling to evaluate the independence and complementarity of TCD relative to established genomic and transcriptomic biomarkers.

In conclusion, this is a post-hoc, hypothesis-generating study investigating whether treatment efficacy differs according to AI-identified TCD phenotype in a sub-cohort derived from a phase III trial comparing two nCRT regimes for patients with LARC. We found that the DFS and OS were improved by the IrCRT in patients with high TCD in the pre-treatment biopsies. These findings suggest that AI-identified TCD is associated with differential outcomes after standard versus irinotecan-intensified nCRT, while warranting independent validation before future use in treatment selection.

## Contributors

MAH and ZS designed the study, with input from the ARISTOTLE trial team (DSM, AL, RB). ZS, MS and CACF developed the AI framework. ZS analysed the data and wrote the manuscript. DB and MAH contributed to the survival analysis and clinical interpretation. NPW contributed to providing the digital pathology data and validating the AI framework. GR contributed to conceptualisation. YZ contributed to data curation. All the authors contributed to the manuscript revision and were involved in the scientific discussion. ZS, CACF, MAH, DB had full access to and verified the integrity of the underlying data. All authors read and approved the final version of the manuscript.

## Data sharing statement

The dataset of the ARISTOTLE trial is available upon request, subject to a signed data access agreement ensuring compliance with ethical guidelines, approval from the institutional review board, and submission of a detailed proposal outlining the intended use of the data. Requests will be considered on a case-by-case basis and should be directed to ctc.aristotle@ucl.ac.uk. The whole AI framework is available via: https://github.com/cacof1/DigitalPathologyAI. The SoftCTM model is available via their own repository https://github.com/lely475/SoftCTM.

## Declaration of interests

NPW is supported by grants from Yorkshire Cancer Research, NIHR, GSK, Pierre Fabre, Roche Diagnostics, and received consulting fees from Bristol Myers Squibb, Astellas, Pfizer, GSK, Amgen, Servier. All other authors declare no competing interests.
