# Supplementary material for: Tumour cell density quantified by artificial intelligence is associated with differential benefit from irinotecan-based chemo-radiotherapy in locally advanced rectal cancer: a post-hoc study of the phase 3 ARISTOTLE trial
Source: eBioMedicine. 2026 Jul 27;130:106397. doi: 10.1016/j.ebiom.2026.106397 (PMC13476900; doi:10.1016/j.ebiom.2026.106397)
Supplement: Supplementary file 1 [file mmc1.pdf]

Tumour Cell Density Quantified by Artificial Intelligence Enables Precise Chemo-Radiotherapy Planning for Patients with Locally Advanced Rectal Cancer: A Post-hoc Study of a Phase III Trial

Zhuoyan Shen*[1], Douglas Brand[1,2], Mikael Simard[1], Andre Lopes[3], Rubina Begum[3], Nicholas P. West[4], Ying Zhang[1], Gary Royle[1], David Sebag-Montefiore[5], Charles-Antoine Collins Fekete[1] and Maria A. Hawkins[1,2]

1. Department of Medical Physics and Biomedical Engineering, University College London, London, UK
2. Department of Radiotherapy, University College London Hospitals NHS Foundation Trust, London, UK
3. UCL Cancer Institute, London, UK
4. Division of Pathology and Data Analytics, Leeds Institute of Medical Research, School of Medicine, University of Leeds, Leeds, UK
5. Division of Oncology, School of Medicine, Leeds Institute of Medical Research, University of Leeds, Leeds, UK

Supplementary Materials

Table S1. Information about the digital pathology data 2
Table S2. Comparison between the whole ARISTOTLE cohort and the subgroup used in this study 2
Table S3. Performance of the AI framework on detecting tumour and stromal tiles. 2

Figure S1. Examples comparing AI-classified tissue types with corresponding manual annotations. 3
Figure S2. Survival difference between TCD-high and TCD-low group in the two treatment arms. a. The Kaplan-Meier curves of DFS (left) and OS (right) of patients who received IrCRT. b. The Kaplan-Meier curves of DFS (left) and OS (right) of patients who received CRT. 4
Figure S3. Survival difference between the two treatment arms in the whole cohort analysed. 5

Table S1. Information about the digital pathology data

Data type	Description
Digitised Whole Slide Images (WSIs)	Digitised WSIs of haematoxylin and eosin (H&E)-stained pre-treatment biopsy (N = 414) obtained from formalin-fixed paraffin-embedded (FFPE) tissue samples. WSIs were sourced from the Virtual Pathology Database at the University of Leeds. All images were scanned at 20× magnification, with a pixel size of 0.5 µm per pixel.<br>Prior to AI analysis, all WSIs were manually reviewed to exclude slides exhibiting significant artifacts and abnormal staining.

Table S2. Comparison between the whole ARISTOTLE cohort and the subgroup used in this study

Variable	Test	Statistic	p
Age	T-test	-0.15	0.8841
Sex	Chi-squared	0.00	1.0000
Treatment Arm	Chi-squared	0.17	0.6786
MRI T stage	Chi-squared	0.36	0.9857
MRI N stage	Chi-squared	1.09	0.9549
DFS	Log-rank test	0.00	0.9906
OS	Log-rank test	0.03	0.8594

Table S3. Performance of the AI framework on detecting tumour and stromal tiles.

Class	Data split	Validation Set			Testing Set		
		Precision	Recall	F1 Score	Precision	Recall	F1 Score
Tumour	A total of 10,000 tiles from the NCT-CRC-HE-100Kdataset were used for training and validation, with 20% allocated to the validation set. An independent hold-out test set (CRC-VAL-HE-7K) comprising 7,180 tiles was used for testing.	0.991	0.989	0.990	0.978	0.964	0.971
Stroma		0.887	0.834	0.860	0.840	0.798	0.818

Table S4. Comparison of Cox proportional hazards models with and without an interaction term between TCD and treatment arm for DFS and OS.

Endpoint	AIC		Log-Likelihood		χ^2	p
	Baseline	Interaction	Baseline	Interaction		
DFS	1936.62	1931.73	-966.31	-962.87	6.88	0.009
OS	1457.47	1448.86	-726.73	-721.43	10.61	0.001

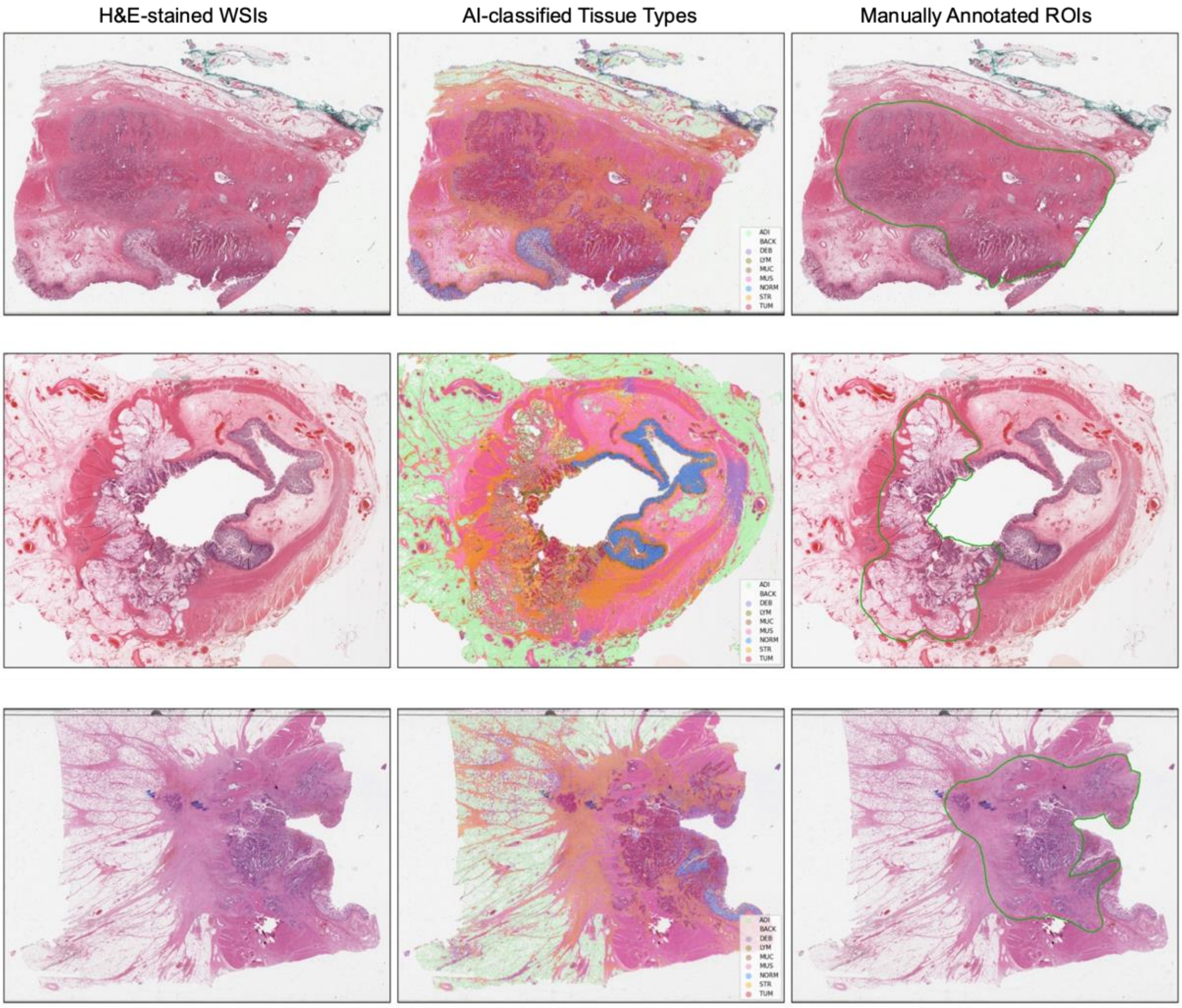

Figure S1. Examples comparing AI-classified tissue types with corresponding manual annotations.

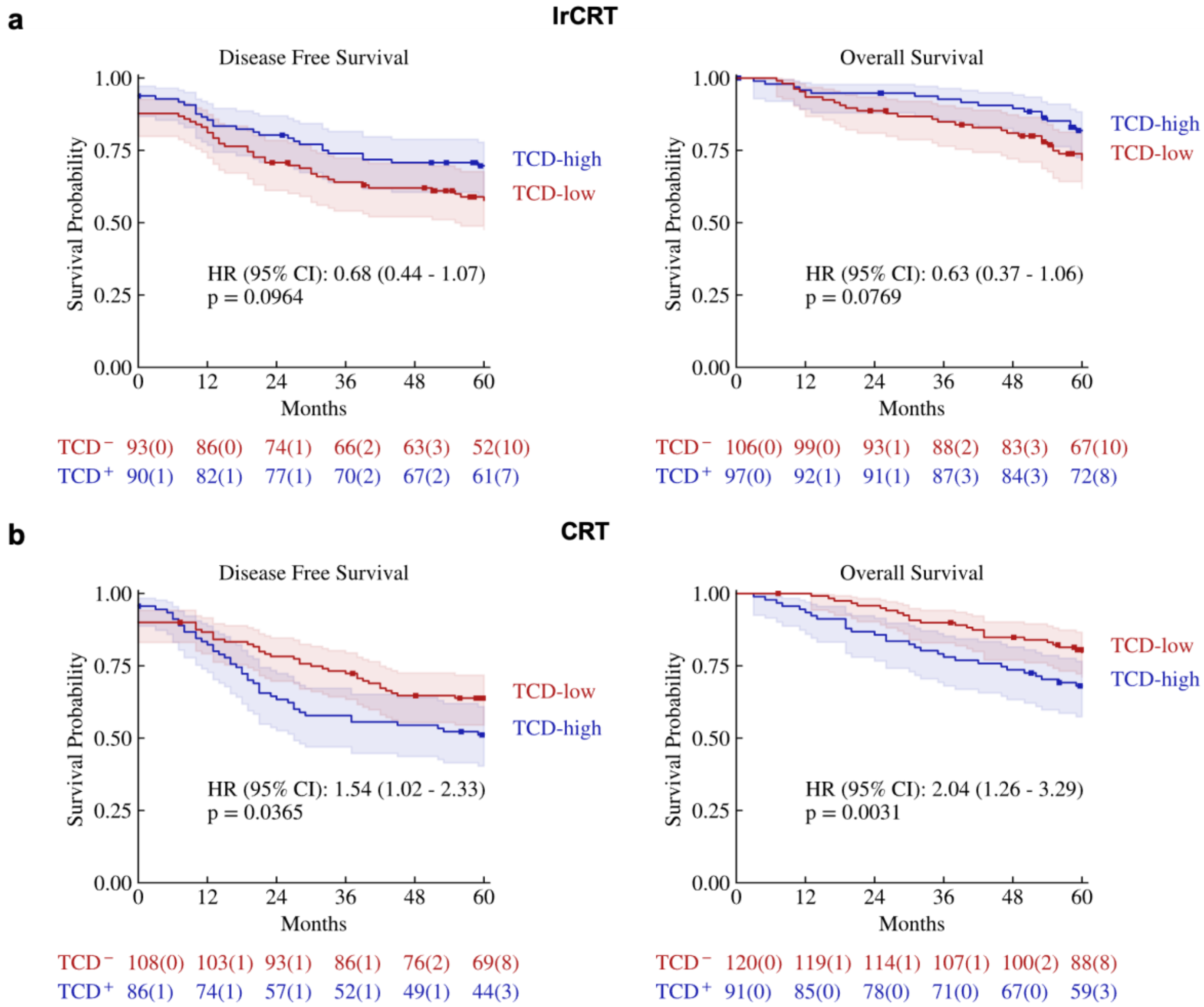

Figure S2. Survival difference between TCD-high and TCD-low group in the two treatment arms. a. The Kaplan-Meier curves of DFS (left) and OS (right) of patients who received IrCRT. b. The Kaplan-Meier curves of DFS (left) and OS (right) of patients who received CRT.

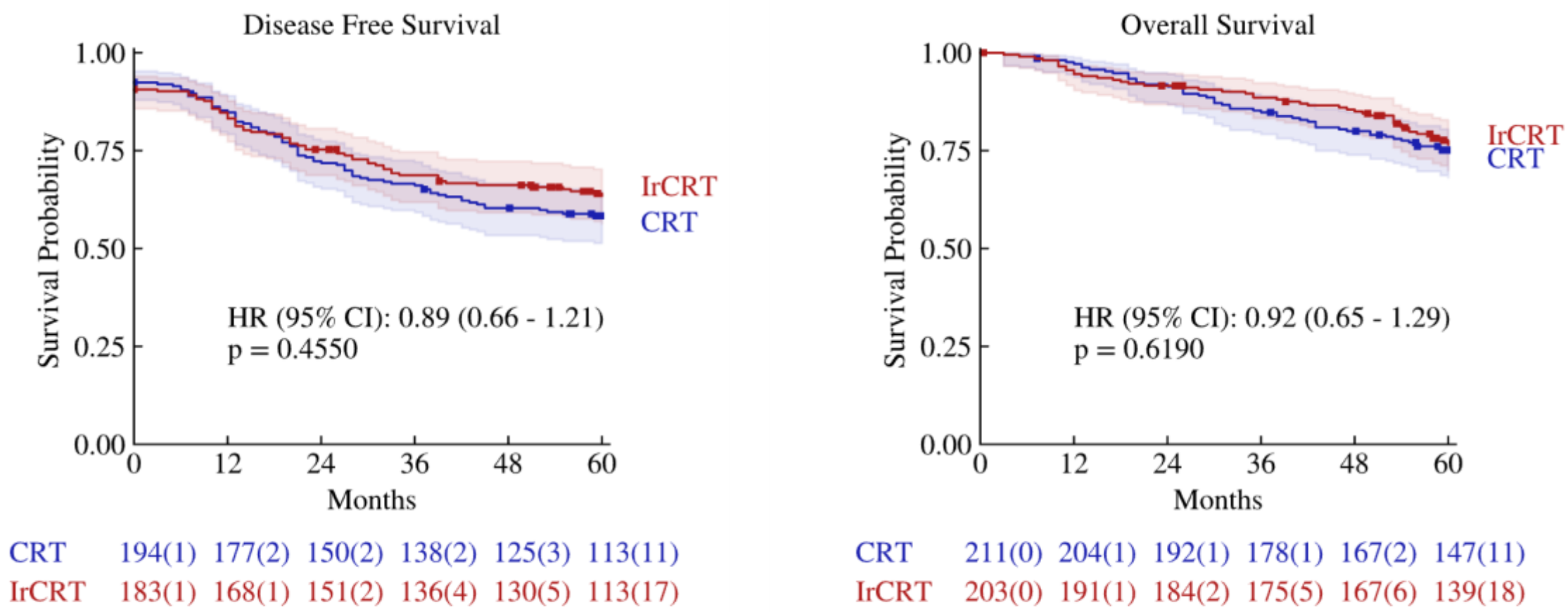

Figure S3. Survival difference between the two treatment arms in the whole cohort analysed.
